# Aspiration of parenteral nutrition – a previously unreported complication of central venous access in an infant: a case report

**DOI:** 10.1186/1752-1947-2-63

**Published:** 2008-02-26

**Authors:** Luke A Jardine, Garry DT Inglis, Mark W Davies

**Affiliations:** 1 Grantley Stable Neonatal Unit, Royal Brisbane and Women's Hospital, Brisbane, Queensland, Australia; 2 Department of Paediatrics & Child Health, The University of Queensland, Brisbane, Queensland, Australia; 3 Visiting Fellow, Medical Engineering, Faculty of Built Environment and Engineering, Queensland University of Technology, Brisbane, Queensland, Australia

## Abstract

**Introduction:**

The insertion of percutaneous central venous catheters is a common procedure in neonatal intensive care nurseries. Placement of the catheter tip in a large central vein is most desirable. Occasionally, due to difficult venous access, catheter tips are left in places that are less than ideal.

**Case presentation:**

A female infant with a complicated gastroschisis developed signs of short bowel syndrome post surgery. She was treated with a combination of parenteral nutrition and enteral feeds. A central venous line was inserted through a scalp vein. The tip was noted to be in a vessel at the level of the mandible. She subsequently became unwell with large milky pharyngeal aspirates and episodes of bradycardia. Chest radiography revealed aspiration. The central venous line was removed because of presumed extravasation. This is the first reported case of parenteral nutrition extravasation into the pharynx causing aspiration in an infant.

**Conclusion:**

This complication may have been prevented by recognising that the tip of the catheter was not correctly placed. When catheters are in unusual positions it may be useful to obtain a second radiograph from a different angle or an ultrasound scan to confirm the positioning of the catheter tip.

## Introduction

The insertion of percutaneous central venous catheters is a common procedure in neonatal intensive care nurseries. Placement of the catheter tip in a large central vein is most desirable. Occasionally, due to difficult venous access, catheter tips are left in places that are less than ideal. Here we present an unusual complication in an infant with a central venous catheter located in a vein of the upper neck.

## Case presentation

A female infant with an antenatal diagnosis of gastroschisis was born by emergency caesarean section for fetal bradycardia at 33^+3 ^weeks post menstrual age. Her 23 year old mother was G2P1, Hepatitis C positive and on Methadone (90 mg daily). Birth weight was 1450 grams.

Initial examination revealed a complicated gastroschisis with a segment of small bowel atresia. The remainder of her clinical examination was otherwise normal. During the first six weeks of life she had three laparotomies with reduction of her abdominal contents, resection of two atretic segments, end to end anastomoses, iatrogenic small bowel perforation, adhesionolysis and the eventual formation of a divided colostomy. Other complications included two episodes of infection in the laparotomy wound requiring intravenous antibiotics. During this period she had two peripherally inserted central venous lines and multiple peripheral intravenous cannulae.

**Figure 1:**
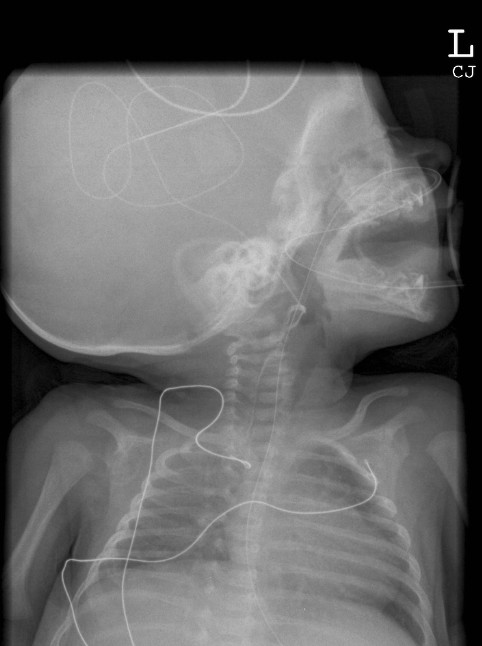
Radiograph post peripherally inserted central venous line insertion.

On day 67 of life, because of short bowel syndrome and intolerance of full enteral feeds of a semi-elemental formula, she was recommenced on parenteral nutrition (120 mL/kg/day) with some nasogastric feeds (60 mL/kg/day). Venous access was extremely difficult to obtain and after two different attempts a 24 French peripherally inserted central venous line (Neocath^®^, Vygon, Ecouen) was inserted through a right sided scalp vein. Blood was easily aspirated from the line at the time of insertion. As per our usual practice, the central venous catheter was slowly injected with 0.5 mL of Isovue 300 (Isovue^® ^300, Regional Health Care Products Group Medi-Consumabales Pty Ltd, Rosebery) and a radiograph was taken while injecting the dye (Figure 1) [1]. The tip was noted to be in a vessel at the level of the mandible and was deemed to be satisfactory for the infusion of parenteral nutrition. The Radiologists report stated the catheter tip location as the "internal jugular vein".

**Figure 2:**
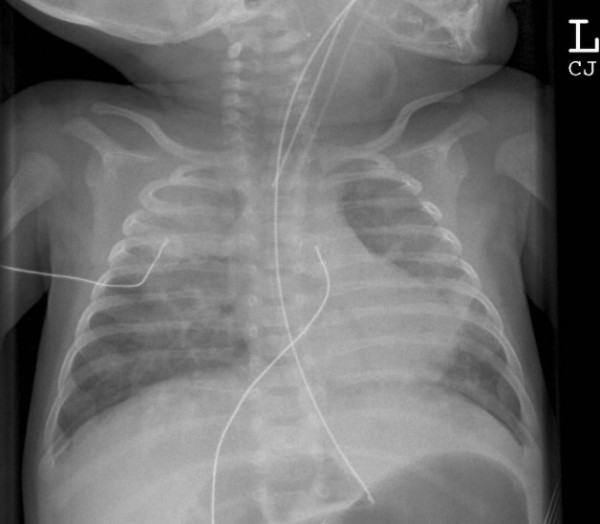
Radiograph taken following episode of aspiration requiring intubation.

On day 80 she became unwell with vomiting and fever. She underwent septic evaluation and was commenced on antibiotics. Enteral feeds were stopped and parenteral nutrition was continued via the central venous line. Over the next 20 hours she became increasingly unwell with worsening vomiting, large milky pharyngeal aspirates and episodes of bradycardia. She required frequent oral suctioning and developed an oxygen requirement. She was intubated and ventilated on day 81. Intubation was difficult with copious milky secretions noted. Chest radiograph showed patchy opacification (Figure 2). Secretions from the oropharynx and nares were so copious that her bedding was wet.

It was suspected that the aspirates contained parenteral nutrition and a sample was sent for microscopy and chemistry. The results were consistent with parenteral nutrition.

On stopping the lipids and parenteral nutrition, a dramatic decrease in the oral secretions was noted and they changed from a milky to clear. The central venous line was then removed because of presumed extravasation. Her condition continued to improve and she was extubated on day 84. She has remained well since extubation apart from ongoing problems with short bowel syndrome.

## Discussion

While extravasation is a recognised complication of central venous catheters, to our knowledge this is the first reported case of parenteral nutrition extravasation into the pharynx causing aspiration in an infant. Frequently reported sites of extravasation include the pleural and pericardial cavities [2,3]. Rarely reported sites of extravasation include the pulmonary parenchyma [4,5], renal pelvis [6], scrotum [7], retroperitoneal space [8], spinal epidural space [9] and subdural space [10].

This complication might have been prevented by recognising that the tip of the catheter was not in the internal jugular vein. It is difficult to accurately locate the catheter tip with a single view.

## Conclusion

When catheters are in unusual positions it may be useful to obtain a second radiograph from a different angle or an ultrasound scan to confirm the positioning of the catheter tip.

## Competing interests

The author(s) declare that they have no competing interests.

## Authors' contributions

All authors contributed to acquisition of case details and the analysis and interpretation of them. LAJ wrote the first draft of the manuscript, GDTI and MWD revised the manuscript. All authors have given final approval of this version to be published.

## Consent

Informed consent was obtained from the patient's parents for publication of this case report and accompanying images. A copy of the consent is available for review by the Editor-in-Chief of this journal.
